# Aptamers: A Feasible Technology in Cancer Immunotherapy

**DOI:** 10.1155/2016/1083738

**Published:** 2016-06-20

**Authors:** M. M. Soldevilla, H. Villanueva, F. Pastor

**Affiliations:** Aptamers Unit, Center for the Applied Medical Research (CIMA), Foundation for the Applied Medical Research (FIMA), Avenida Pío XII No. 55, 31008 Pamplona, Spain

## Abstract

Aptamers are single-chained RNA or DNA oligonucleotides (ODNs) with three-dimensional folding structures which allow them to bind to their targets with high specificity. Aptamers normally show affinities comparable to or higher than that of antibodies. They are chemically synthesized and therefore less expensive to manufacture and produce. A variety of aptamers described to date have been shown to be reliable in modulating immune responses against cancer by either blocking or activating immune receptors. Some of them have been conjugated to other molecules to target the immune system and reduce off-target side effects. Despite the success of first-line treatments against cancer, the elevated number of relapsing cases and the tremendous side effects shown by the commonly used agents hinder conventional treatments against cancer. The advantages provided by aptamers could enhance the therapeutic index of a given strategy and therefore enhance the antitumor effect. Here we recapitulate the provided benefits of aptamers with immunomodulatory activity described to date in cancer therapy and the benefits that aptamer-based immunotherapy could provide either alone or combined with first-line treatments in cancer therapy.

## 1. Introduction

### 1.1. Challenges in Cancer Therapy

Cancer is a malignant disease caused by an abnormal noncontrolled cellular growth which acts autonomously and is capable of invading either local or distant tissues. Cancer cells are known to show common special features acquired during cancer development. These features include sustained proliferative signaling, evading tumor suppressors, resisting cell death, enabling replicative immortality, inducing angiogenesis, and activating invasion and metastasis [1, 2]. In fact, more than one and a half million new cancer cases were estimated only in the US in 2016 and almost six hundred thousand of these were estimated to have died of cancer [3]. Moreover, the cancer burden around the world is alarmingly growing, so that about twenty-one million of new cancer cases and thirteen million of cancer deaths are expected to occur from now until 2030 [4]. The first-line treatments nowadays against cancer are chemotherapy, radiotherapy, and surgery when tumor is resettable. Current conventional cancer treatments are usually not enough in advanced aggressive tumors, as it is reflected in the high number of relapsing rates and the high toxicity associated with current treatments due to their lack of specificity. Surgery has the intrinsic technical problem of not removing every single malignant cell, which causes tumor relapses in the majority of the cases, and chemotherapy and radiotherapy are very harmful and usually show serious side effects. Due to this limited success of chemotherapy and radiotherapy, new alternative and more specific treatments are strongly needed. There is an urge to identify additional treatments to improve patient's survival and reduce toxicity. Immunotherapy has emerged as the most feasible alternative thanks to its high tumor specificity in comparison with chemotherapy. Besides the recent encouraging results obtained in clinical trials during the last years [5], followed by the FDA approval of several immune-checkpoint-blockade monoclonal antibodies (mAbs) [6–11], have pushed cancer immunotherapy back to prominence. The use of these monoclonal antibodies, either as monotherapy or in combination, has achieved very significant results in different kinds of cancer [12]. Nevertheless, the use of immunomodulatory monoclonal antibodies has been associated with severe toxicities [13–16]. The 4-1BB Ab therapy has been correlated with hepatic toxicity and TGN1412 (CD28 superagonistic monoclonal antibody) caused a lethal cytokine storm [13].

Targeting specific immunomodulatory ligands at the tumor site would increase the antitumor effect of the given therapy and would decrease the severe toxicities associated with dose-limiting and off-target side effects in patients, thereby widening the therapeutic index. Thus, in order to fine-tune the antitumor treatments, it is of great importance to find a clinically feasible tool to that effect. Several approaches with different protein-derived products, such as engineered antibodies, diabodies, and chimeric receptors, have been described to this end [17–21]. Within the last few years, aptamers have appeared to be a promising platform for targeted cancer therapy [22].

### 1.2. Aptamer Development/SELEX

**Figure 1:**
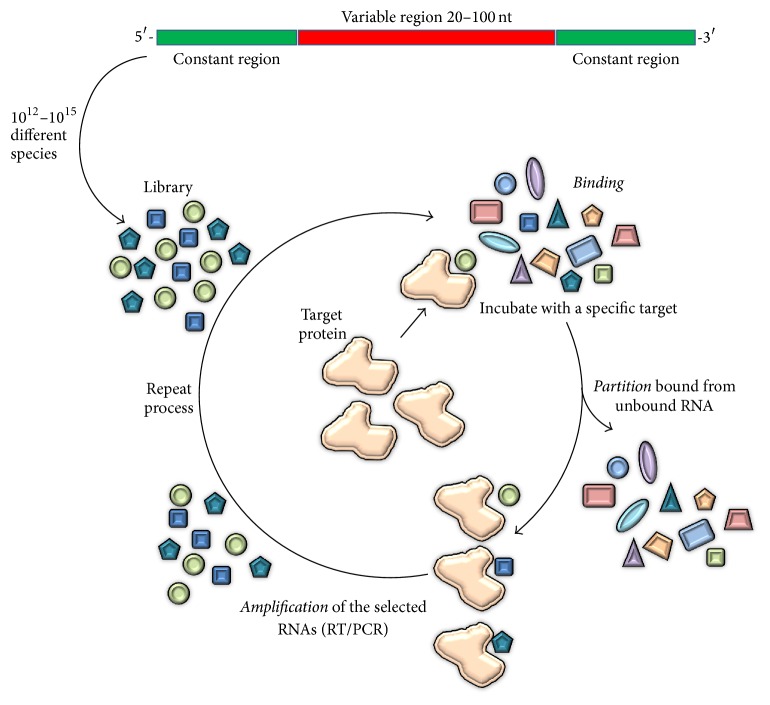
Schematic representation of SELEX procedure.

Aptamers are single-stranded DNA or RNA oligonucleotides (ODNs) with a tridimensional folding that confers on them a high specificity and affinity for their targets. Aptamers are selected by a process called SELEX. The word SELEX means Systematic Evolution of Ligands by Exponential Enrichment. The SELEX procedure was developed by Tuerk and Ellington [23, 24] in the early 90s and consists in rounds of iterative selection [23, 24]. As can be seen in Figure 1, each round comprises basically three steps: binding, partition, and amplification. In the first step (binding), a library is incubated in the presence of the target. This library consists of a random sequence which can vary between 20 and 100 nucleotides (nt) flanked by two constant regions at 5′ and 3′ end. For the partition step, the bound sequences are removed and target-bound sequences are separated from the target. Finally, the target-binding species are amplified by polymerase chain reaction (PCR) using primers that anneal at the two constant regions. If the desired aptamer is an RNA aptamer,* in vitro* transcription shall be performed before starting the next round. The selection procedure may usually vary between 9 and 12 rounds of selection, which implies months of work. Nowadays, techniques such as high-throughput sequencing which allow us to detect already sequence-enriched families favor the selection procedure drastically reducing the number of rounds [25, 26]. The SELEX procedure has evolved since the first SELEX protocol new variation of the selection procedures emerged. Some of the now used types of selection procedures besides conventional SELEX are CE-SELEX, which come from capillary electrophoretic SELEX [27]; cell-SELEX, in which selection is carried out with cells [28]; or toggle-SELEX, which on the other hand is used to obtain cross-reactive aptamers [26, 29]. A new class of aptamers has been recently described, which are “mirror-image” L-conformed enantiomer aptamers and are called Spiegelmers [30]. A novel variation of conventional SELEX known as Tailored-SELEX [31] is used to identify ten fixed nucleotide aptamers and no primer binding sites. This approach was validated by identifying a Spiegelmer against the migraine-associated target calcitonin gene-related peptide 1 (alpha-CGRP) [31]. Moreover, the selection procedure which performs the rounds in animals is called* in vivo* SELEX [32], and genomic SELEX is otherwise used to achieve genomic-encoded functional domains, known as genomic aptamers capable of binding to a specific target [33]. This great variety in aptamer selection procedures allows the technique to effectively isolate aptamers against almost every target and of almost any nature. Aptamers can indeed be generated against most targets, including proteins and even small molecules [22, 34].

The SELEX (Systematic Evolution of Ligands by Exponential Enrichment) procedure is an iterative selection of single-stranded oligonucleotides that bind to specific targets. This technique consists of selection rounds which in turn are divided into three steps: binding, partition, and amplification. The first round starts with a randomized library which is incubated with the target during the binding step. Afterwards, nonbinding species are discarded and the resulting binding species are separated from the target during the partition step. Finally, the binder sequences are amplified by PCR and prepared for the next round. It is noteworthy that if the aptamer of interest is an RNA aptamer,* in vitro* transcription shall be performed before starting the new round.

Aptamers are synthetic ODNs known by their avidity for their cognate target. The affinity and specificity are in the majority of cases comparable or even superior to those of antibodies. A DNA aptamer that recognizes its target with a dissociation constant (*K*_*d*_) of 0.2 nM has been recently described [38]. As mentioned before, aptamers possess several advantages when compared with cell-based products such as antibodies or recombinant proteins. In contrast to what might occur with antibodies, the toxicity or low immunogenicity of specific antigens does not interfere in the aptamer selection process [50]. Regarding translation into the clinic, aptamers provide the advantage over antibodies of presenting inherently an antidote [51, 52]. The presence of a universal antidote ensures the reversion of its activity if any undesirable effect would arise. Short ODNs show lack or much reduced immunogenicity when compared to antibodies due to the absence of T cell-dependent immunity. Moreover, aptamers can be easily modified by chemical processes so as to optimize yield and provide custom-tailored properties. Plasma stability can also be significantly enhanced by selective* O*-methyl or F substitutions for OH residues at the 2′ position of the bases [22, 53]. In order to widen the sort of aptamer interactions with their targets that can be achieved by including other nonnatural analogous bases [22, 53], there is the addition of aromatic hydrophobic modified nucleotides such as benzyl-dU (Bn-dU) and naphthyl-dU (Nap-dU) as in the case of SOMAmers [54]. SOMAmers (slow-off rate modified aptamers) show protein-like modified side chains. These modifications confer on them the advantage over regular aptamers of exposing fewer hydrogen bonds, fewer charge-charge interactions, and fewer total polar contacts as determined by the summatory of hydrogen bonds and charge-charge interactions [54]. To increase the effective molecular size, conjugation of aptamers to cholesterol led to a significant enhancement of the* in vivo* half-life of the ODNs in mice accompanied by a dramatic improvement of biological activity [55–57]. Another alternative, clinically compatible carrier is polyethylene glycol (PEG) [58], which prevents its exclusion by renal filtration [22]. Such postselection modification increases aptamer survival to the maximum rates of sixfold as it has been recently attributed to a PEGylated anti-MUC1 aptamer-doxorubicin conjugate [59].

Thanks to their small size, aptamers can easily penetrate tissues, which favors cell targeting. In addition, aptamers can easily be modified to add custom-tailored properties. Aptamers can be engineered to be multimerized to modulate the immune system [35, 42, 45, 46, 50] or to carry very different cargoes such as drugs, radioisotopes, proteins, enzymes, RNAs, or even nanostructures [60–62]. They can be chemically synthesized and therefore easy to export to good manufacturing practices (GMP) grade. GMP are the summation of practices required to manufacture and sell active pharmaceutical products in this specific case. These guidelines provide the minimum requirements that a pharmaceutical manufacturer must meet to assure that the products are of high quality and do not pose any risk to the consumer or the public. Unlike aptamers, antibodies are hindered by several factors at GMP grade. They are cell-based products and that toughens the regulatory approval process and increases the complexity and cost of manufacturing [50].

Since the first aptamer isolated in the early 90s, a tremendous amount of new selected aptamers has been published [22, 63, 64]. Some of them are currently undergoing clinical trials for the treatment of several diseases. The anti-PDGF (platelet-derived growth factor) and the anti-C5 (complement component 5) RNA aptamers are used to treat macular edema and age-related macular degeneration [65, 66]. The antifactor IXa of coagulation and the anti-A1 domain of activated von Willebrand factor (vWf), among other RNA aptamers, are aimed at controlling hemostasis [66–68]. For the treatment of diabetes mellitus, one Spiegelmer aptamer that targets the monocyte chemoattractant protein 1 (MCP-1 also called CCL2) is used [66]. The antinucleolin aptamer AS1411 and the antistroma cell-derived factor-1 (SDF-1 also called CXCL12) NOX-A12 are among the most advanced aptamers for cancer treatment [66]. The first in class was the anti-VEGF RNA aptamer approved in 2004 by the FDA, called MACUGEN, and used for the treatment of age-related macular degeneration. Despite the fact that plenty of aptamers have been described in every research field and some of them are used in cancer therapy, in this review we focus on those aptamers used in cancer immunotherapy.

## 2. Aptamer-Based Immunotherapy

As was mentioned above, there is an urge to develop new therapeutic approaches that synergize with conventional chemotherapy and prolong the antitumor effect. We can also envision that the field of cancer immunotherapy is leading towards a multipronged approach to tackle the tumor at different fronts. Cancer immunotherapy has come to prominence upon the success of recent clinical trials with immune-checkpoint blockade. The immune system is well educated but dysfunctional.

Lack of costimulation and antigenicity as well as presence of immune-suppressor factors within the tumor microenvironment favors immune escape. Thus, major challenges in cancer immunotherapy are (I) to activate the immune system to attack the tumor triggering costimulatory signals within the tumor, (II) to counteract negative signals to favor the action of the immune system in the tumor microenvironment, and (III) to enhance tumor antigenicity by the expression of potent tumor neoantigens. Following this line, cancer can be attacked from different flanks by pushing the accelerating pedals and releasing the brakes at the same time. Moreover, lack of tumor antigenicity is a major bottleneck for the success of immune-checkpoint-blockade antibodies, as it has been recently published [69–71]. A feasible approach to induce the expression of new tumor antigens has been described by Pastor et al. [72] by controlling the NMD inhibition in the tumor which allows for the expression of mutated tumor antigens with premature stop codons that are usually blocked by this mechanism [72].

To date, numerous aptamer constructs have been described that are able to modulate the immune response against cancer [50]. They provide a similar or even superior activity to that of the corresponding monoclonal Ab, and their superior targeted delivery capacity confers on them less off-target side effects [73]. Thanks to their plasticity, aptamers are a very promising tool as immune-modulatory ligands, since they can be engineered to either activate or block an immune-modulatory receptor [45, 46, 50]. They can be customized to target this immunomodulation to the tumor site and can be engineered for delivering almost any kind of cargo as well [22].

### 2.1. Antagonistic Aptamers in Cancer Immunotherapy

**Figure 2:**
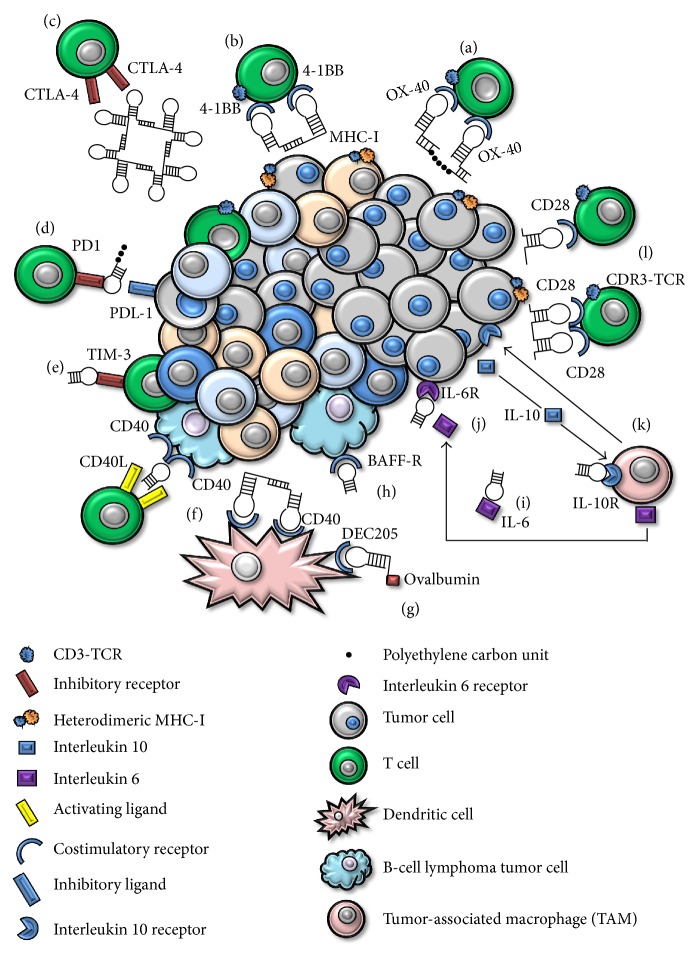
Schematic representation of main aptamers used in cancer immunotherapy. (a) OX-40. (b) 4-1BB. (c) CTLA-4. (d) PD1. (e) TIM-3. (f) CD40. (g) DEC205. (h) BAFF-R. (i) IL-6R. (j) IL-6. (k) IL-10R. (l) CD28.

In 2003 the development of first immune-checkpoint-blockade RNA aptamer that binds CTLA-4 was published by Gilboa's group [35]. The selection of these anti-CTLA-4 aptamers was the first being used with immunotherapeutic intentions and opened the door to a new platform in cancer immunotherapy. The CTLA-4 aptamer bound to its target with high affinity and specificity. The aptamer was multivalently engineered (schematically represented in Figure 2(c)) and these aptamers showed inhibition of CTLA-4 function* in vitro* and enhancement of tumor immunity in mice. Moreover, this assembling of the aptamers into tetrameric forms significantly enhanced their bioactivity* in vitro  *and*  in vivo*. CTLA-4 aptamer showed a similar effect to that of the mAb. Indeed, a recently published work demonstrates that CTLA-4 delivery strategies are able to target CD8^+^ infiltrated lymphocytes and regulatory T lymphocytes (Tregs). This CTLA-4 aptamer-based targeting delivery of STAT-3 siRNA to T lymphocytes resulted in inhibition of tumor growth and of metastasis [74]. STAT-3 promotes tumor-cell survival and proliferation in tumor cells, as well as invasion and immunosuppression [74]. It has been widely demonstrated how STAT-3 inhibition can be targeted by TLR9 natural ligands such as CpG. This targeted inhibition leads to activation of tumor-associated immune cells and strong antitumor immune responses [75, 76]. Moreover, STAT-3 is persistent in immunosuppressive cells and contributes to the expansion of CD4^+^ Tregs. In the work presented by Herrmann et al. [74] they show an increase of CD8^+^ T-effector response* in vivo* due in the first place to the blockade of CTLA-4 and subsequently to the STAT-3 silencing. Silencing STAT-3 provided a systemic antitumor response downregulating CD4^+^ Tregs which was reflected in inhibition of tumor growth in various cancer cell lines and metastasis too [74].

PD1 is expressed in several cell types including T cells, specifically in CD8 tumor-infiltrating lymphocytes (TILs) which are in charge of directly eradicating tumor cells [77]. The engagement with PD1 expressed on the surface of lymphocytes within the tumor microenvironment and PDL-1 expressed on tumor cells leads to lymphocyte dysfunction by T cell exhaustion and tumor progression. It has recently been published that a DNA aptamer, represented in Figure 2(d), aimed at blocking the PD1 receptor decreases tumor growth and increases survival in mice tumor models [37].

Another exhaustion-associated T cell receptor is TIM-3, which is coexpressed with PD1 by exhausted and dysfunctional T cells [36]. TIM-3 has been also identified in a subpopulation of regulatory T cells with a potent immune-suppressive activity which correlates with a bad prognosis in cancer patients [36]. Recently our team has published a TIM-3 RNA antagonistic aptamer (shown in Figure 2(e)) able in a therapeutic setting to synergize with suboptimal doses of PDL-1 blockade [36]. This strategy led to an important antitumor activity* in vivo* at very low doses of both TIM-3 aptamer in its monomeric form and PDL-1 blocking antibody [36].

Furthermore, several aptamers have been described in an immunotherapy context towards some cytokine blockade. An aptamer known as R5A1 that binds to IL-10R has been selected and optimized to block the interaction between IL-10 and its receptor on the surface of immune-system cells. IL-10 is known to be secreted by tumor cells and promote immune-modulatory responses that favor tumor establishment and growth [25]. The aptamer bound to IL-10 receptor on the cell surface and blocked IL-10 function* in vitro*. Moreover, the aptamer sequence and therefore the structure were optimized by truncation, discarding putative steric domains increasing aptamer affinity. In addition, systemic administration of the aptamer was capable of inhibiting tumor growth in mice to a comparable level of that of an anti-IL-10 receptor monoclonal antibody [25]. The tetrameric form of R5A1 aptamer blocked the IL-10 signaling* in vitro *and the systemic administration of the truncated 48-nucleotide (schematic representation in Figure 2(k)) long* in vivo *blocked IL-10, an action that resulted in inhibition of tumor growth [25]. Moreover, an antagonist aptamer against both human and murine IL-RA has been recently published by using high-throughput sequencing (HTS). This work describes a parallel murine and human specific target selection (IL-10RA and 4-1BB) followed by identification of common sequences. This “toggle-type” SELEX allows for the selection of cross-reactive species in a very feasible manner [26]. These aptamers and the majority of them used to date in cancer immunotherapy are summarized in Table 1.

Furthermore, two SOMAmers that bind to IL-6 have been lately selected [38]. IL-6 is an immune-suppressive cytokine produced by B cells, T cells, monocytes, and fibroblasts, among other cell types. IL-6 secretion by immune cells within the tumor microenvironment leads to accumulation of regulatory cells. These proinflammatory conditions favor immune regulation and disrupt the balance towards tumor growth. Thus, the selection of aptamers that block the IL-6 action has been of great interest. Both selected SOMAmers prevent IL-6 signaling by blocking the interaction of IL-6 with its receptor (schematically represented in Figure 2(i)) and inhibit the* in vitro* proliferation of tumor cells at levels of efficacy comparable to those of the anti-IL-6 mAb tocilizumab [38].

Another promising strategy is the specific targeting inhibition of interleukin-6 receptor (IL-6R) and delivery through this molecule. A specific IL-6R RNA aptamer has been described that recognizes its target with high affinity, as represented in Figure 2(j). This aptamer showed no blocking activity between IL-6R and its natural ligand. Nevertheless, it was able to specifically internalize and deliver cargoes to IL-6R expressing cells [39].

More aptamers have been selected against other cytokines. A 2012 publication showed that a human and murine RNA cross-reactive aptamer against the interleukin 4 receptor alpha was able to trigger apoptosis in myeloid-derived suppressor cells (MDSCs). This approach targeted MDSCs and tumor-associated macrophages (TAMs) which displayed an increased number of tumor-infiltrating lymphocytes (TILs) and a reduction in 4T1 mammary carcinoma murine tumor model [40]. In the same year, Orava et al. [41] described a human DNA antagonistic aptamer against the tumor necrosis factor alpha (TNF-*α*) capable of blocking its activity* in vitro* [41].

BAFF-R causes proliferation and cell survival upon ligand engagement favoring tumor growth, which enables tumor cells to grow faster than nonmalignant B cells. Aptamers that bind BAFF-R have been selected and showed to inhibit BAFF-mediated proliferation and survival of malignant B cells [49]. Antagonistic aptamers (as represented in Figure 2(h)) for BAFF-R have been selected and demonstrated to inhibit malignant B-cell proliferation [49].

Following this line we have recently published a murine aptamer against CD40 as a monomer that acts as a CD40 antagonist, thus blocking the downstream signaling (Figure 2(f)). The CD40 aptamer blockade which is expressed in several B-cell malignancies reduces tumor growth and augments mice survival by 30% [46].

Moreover, we have described a CD28 antagonist aptamer (represented in Figure 2(l)). This aptamer is capable of competing for CD28 receptor with its natural ligand B7, as was shown* in vitro *in proliferation assays [45]. In these experiments the antagonistic aptamer was able to revert* in vitro* the costimulation in CD4^+^ T cells induced by B7 ligand [45]. This approach of blocking CD28 would be of great interest in autoimmune diseases or transplants. During transplants acute host versus donor immune responses are developed upon engraftment and immune-suppressor drugs need to be administered to the patient in order to suppress these acute responses. Administration of this CD28 antagonist aptamer would suppress immune responses driven by activated T lymphocytes facilitating donor engraftment.

Various Spiegelmers have been shown to be very effective in animal models. Two Spiegelmers against CCL2 (NOX-E36) and CXCL12 (NOX-A12) have undergone regulatory safety studies demonstrating good safety profiles in healthy volunteers and today are under Phase IIa studies in patients [66]. Moreover, a human and murine cross-reactive Spiegelmer named NOX-D20 has been described that specifically antagonizes the complement component C5a. This L-conformed aptamer prevents organ failure and improves survival in a model of sepsis as well as suppressing local and systemic inflammation [78].

### 2.2. Agonistic Aptamers

Activating the positive signals, which corresponds to pushing the gas pedal, has been and remains one key strategy in cancer immunotherapy. Apart from the activating signal which comes from the coengagement of MHC-I, TCR, and CD3, a second signal known as second costimulatory signal is required for the proper T cell activation. The tumor microenvironment usually lacks costimulatory ligands such as CD80 or CD86. This lack of costimulation leads CD8^+^ T cells to become anergic and therefore unable to trigger an immune response. Several selected aptamers to major costimulatory receptors (4-1BB, OX-40, or CD28) have been selected and engineered to costimulate T cells. Costimulation of T lymphocytes with ODN aptamers has been already well documented to date [42, 45, 79, 80]. Nevertheless, it was not until 2008 that the first aptamer directed to a costimulatory receptor was selected, optimized, and engineered, as represented in Figure 2(b) for costimulation. This CD8^+^ T cell costimulation was shown to inhibit tumor growth in murine models [42]. 4-1BB is expressed on T lymphocytes and is one of the major costimulatory receptors that promotes survival and expansion of activated T cells. Its ligand 4-1BBL is expressed on professional antigen-presenting cells (APCs) such as dendritic cells (DCs) [42]. That work, published by Gilboa's group [42], strengthened the idea of using aptamers in cancer immunotherapy. Moreover, beyond this murine aptamer, as mentioned above, a murine and human cross-reactive RNA aptamer has been described [26].

OX-40 is a costimulatory receptor expressed on CD4^+^ cells upon TCR activation. The engagement with its natural ligand OX-40-L expressed on APCs leads to T cell proliferation, increased cytokine release, and T-lymphocyte survival [43]. OX-40 aptamers with immunotherapeutic intention were published in the year 2008 reinforcing this new platform in cancer immunotherapy. This aptamer was assembled in a two-copy scaffold to yield the costimulatory effect [44]. These two artificial costimulatory aptamers (4-1BB and OX-40) were engineered by two different ways. 4-1BB dimers were obtained by adding short complementary sequences at 3′ ends which will anneal by pairwise fashion (schematic representation in Figures 2(a) and 2(b)). On the other hand, the OX-40 dimer was generated by adding an 18 carbon-length polyethylene spacer between the two 3′ aptamer-end complementary sequences that will anneal by pairwise fashion [42, 44]. In addition, an agonistic aptamer towards human OX-40 has been selected and shown to be dimerized to exert its costimulatory effect, which was mirrored in cellular proliferation and increased INF-gamma production [44].

CD28 is known to be a very important receptor in an immunotherapy context. CD28 is one of the main costimulatory receptors. T cells need at least two signals to be properly activated, as mentioned above, and the most important costimulatory signal comes from the engagement of CD28 and its natural ligand B7 (CD80 and CD86). Various aptamers for targeting and costimulating CD28 have been engineered and they have shown a more potent costimulatory effect than that of the mAb 37.51 [45]. In this work published by our group, dimerization was achieved as mentioned above by adding short sequences to the 3′ end that will anneal by pairwise fashion, displaying more flexibility and mirroring the average distance between the 2 Fv of an IgG and guaranteeing a more rigid structure. Nevertheless, the highest effect was achieved when generating the dimer by PCR, resulting in a shorter linker, which reduces the distance to the minimum and provides flexibility probably responsible for its augmented costimulatory capacity. Any of the dimeric constructs comprised the affinity for its target indicating that the length and flexibility of the linker could be modified to enhance the costimulatory effect. Finally, and not less importantly, this work described for the first time a single molecule with the dual role of acting as antagonist in its monomeric form and as agonist by simple dimerization [45]. The main constructs of CD28 agonistic aptamer among others dedicated to costimulating T lymphocytes are schematically represented in Figure 2(l). Furthermore, an idiotype vaccination context triggered a cellular and humoral antitumor immune response more potent than that of the anti-CD28 mAb. This humoral and cellular response was reflected in both tumor growth and survival [45].

As has been recently published by our group, two agonistic CD40 aptamer-based constructs are able to recover bone-marrow aplasia while increasing antigen-presenting cells (APCs) activation [46]. One of the aptamers described in this work acts as an antagonist, as mentioned above, and again by engineered dimerization we were able to turn it into an agonist. We describe two different CD40 agonistic aptamer constructs able to activate APCs displaying an increased proliferation and expression of costimulatory ligands [46]. The main constructs of these RNA aptamers are represented in Figure 2(f).

Recently a DEC205 RNA murine aptamer able to induce specific antigen cross-presentation has been described (represented in Figure 2(g)) [47]. DEC205 is a surface protein expressed mostly in CD8*α* ^+^ dendritic cells. DEC205 displays antigen cross-presentation and the subsequent CD8^+^ T-lymphocyte activation [47]. This aptamer-based approach is shown to be efficient for both* in vitro *and* in vivo* delivery of specific cargoes for cross-priming. This work demonstrated the potential of this strategy by strongly enhancing T cell-mediated antitumor immunity [47].

**Figure 3:**
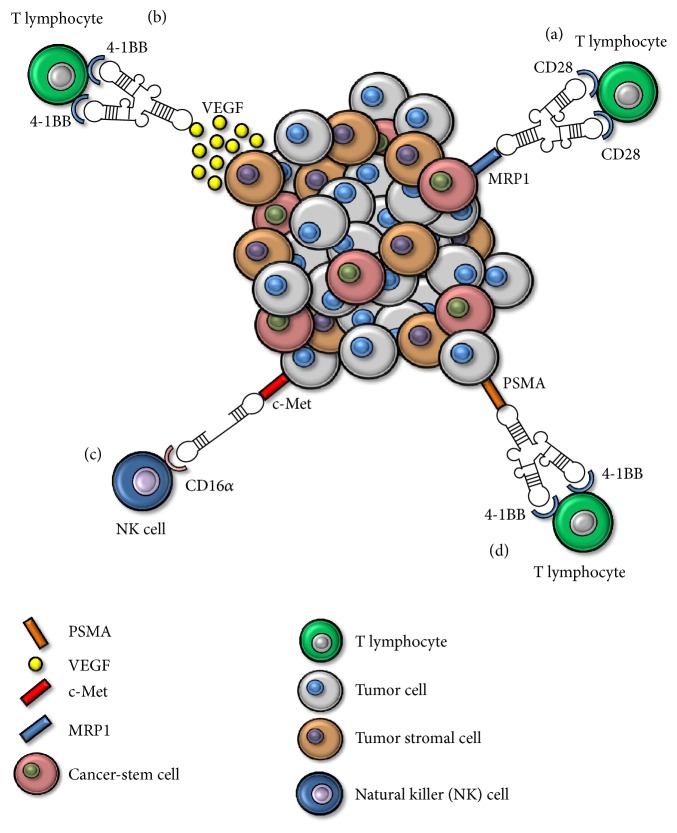
Bispecific aptamers used to date in cancer immunotherapy. (a) Bispecific aptamer CD28-MRP1. (b) Bispecific aptamer 4-1BB-VEGF. (c) Bispecific aptamer CD16*α*-c-Met. (d) Bispecific aptamer 4-1BB-PSMA.

Finally, a DNA aptamer against the Fc*γ* receptor III (CD16*α*) was developed to generate a bispecific aptamer to target the antibody-dependent cell-mediated cytotoxicity (ADCC) to c-Met overexpressing tumor cells, as represented in Figure 3(c) [48]. This bispecific aptamer was demonstrated to elicit specific ADCC in both human gastric and lung cancer cell lines [48].

#### 2.2.1. Overriding Immune Therapy Toxicity: Targeting Costimulation to the Tumor

The use of agonistic antibodies often leads to off-target toxicity, as happened with some agonistic antibodies described to date with 4-1BB or CD28. Targeting costimulation to the tumor site would decrease these side effects, increasing the therapeutic index. Immune-checkpoint mAbs-based therapy has shown several adverse side effects, such as severe hepatotoxicity, lymphopenia, and thrombocytopenia [81]. The administration of agonistic CD28 antibodies such as TGN1412 displayed multiorgan failure [13] and made the end of the clinical trial necessary. Liver toxicity is one of the major concerns in 4-1BB mediated treatment of cancer [14]. In order to increase the therapeutic index, the first bispecific aptamer was generated [82]. Bispecific aptamers consisting of both PSMA-4-1BB aptamers have been engineered (schematically represented in Figure 3(d)) and showed potent antitumor effects even in low doses when compared with nontargeted costimulation and the correspondent mAbs controls. Lower levels of PSMA-4-1BB bispecific costimulation were nearly as effective as tenfold increased levels of nontargeted costimulation or the corresponding monoclonal antibody [82]. Therefore, as demonstrated by Pastor et al. [82], targeting 4-1BB costimulation to the tumor site requires less amount of reagent to achieve a therapeutic effect, exhibiting a superior safety profile [82]. Moreover, a work has been recently published in which 4-1BB costimulatory aptamer is directed to tumor stroma through the VEGF aptamer (represented in Figure 3(b)). This bispecific aptamer has again shown a higher therapeutic index in comparison with the nontargeted costimulation reagents, reaching the same effect with less toxicity [79]. This increase in therapeutic index reduced CD8^+^ T cell hyperplasia as well as spleen, lymph node, lung, and liver weights while displaying a similar antitumor effect [73]. Other aptamers such as OX-40 and CD28 have also been shown to be equal to or more potent than their respective mAbs [35, 42, 43, 45]. Thus, targeting costimulation to the tumor site seems to be a feasible strategy in antitumor treatments. Furthermore, targeting costimulation towards markers implicated in tumor-chemotherapy resistance would act exerting a selection pressure against these types of tumor cells. It is known that cancer-stem cells are responsible for tumor metastasis, chemotherapy resistance, and tumor relapses [83, 84]. We have selected a MRP1 aptamer by using a novel combinatory peptide-cell HT SELEX method [80]. We used this aptamer to isolate a subpopulation of chemotherapy-resistant MRP1-expressing melanoma cell line. We then used this aptamer to generate a new CD28-MRP1 bispecific aptamer, as represented in Figure 3(a). This bispecific CD28-MRP1 aptamer was able to target* in vitro *and* in vivo *costimulation to MRP1-expressing cells and capable of providing a proper costimulatory signal to T cells [80]. In combination with Gvax and the FOXP3 temporary inhibitor peptide P60 the bispecific aptamer induced higher T cell tumor infiltration, slower tumor growth, and longer survival [80]. We also developed a new strategy to coat* ex vivo* MRP1-expressing tumor cells to create a new vaccination approach named CD28Aptvax. This vaccine consists in irradiated B16-MRP1 cells coated with the bispecific aptamer and its administration in mice significantly delayed MRP1-expressing tumor growth and maintained around 50% survival after 50-day follow-up [80]. The summary of bispecific aptamers is represented in Figure 3.

### 2.3. Enhancing Tumor Immunogenicity

Despite the efforts invested in potentiating tumor immunity by activating costimulatory receptors with agonists or inhibiting undesired immunosuppressive responses, absence of antigenicity is a problem that remains unresolved. The enhancement of tumor immunogenicity is one of the most difficult challenges to face in cancer immunotherapy. One of the strategies described to date is to increase the tumor antigenicity by expressing new antigens. The Nonsense Mediated mRNA Decay (NMD) is in charge of deleting mRNAs that encode premature termination codons (PTCs). NMD inhibition induces the expression of new products in the cells [85]. Moreover, the correlation between NMD inhibition and lymphocyte infiltration has been demonstrated [86]. In that work published by El-Bchiri et al. [86], higher accumulation of CD3^+^ cells was inversely correlated with NMD function in colorectal cancer with microsatellite instability (MSI). Thus, targeting the siRNA-mediated inhibition of NMD in tumor cells as was already published indeed induced an antitumor immune-system mediated response [72]. A chimera consisting in PSMA aptamer coupled with NMD factor-siRNAs resulted in an increase of tumor antigenicity* in vivo* reducing tumor growth [72]. Given the fact that activating CD40 signaling would promote CD40-expressing tumor progression, we managed to generate a CD40 agonistic aptamer conjugated with a shRNA aimed at inhibiting NMD [46]. As mentioned above [72, 85, 86], NMD inhibition leads to the expression of new and therefore more potent antigens that renders tumor regression. This optimized chimera was capable of enhancing tumor antigenicity leading to increased lymphocyte infiltration, lowering tumor growth in a B-cell lymphoma tumor model, and increasing mice survival [46]. This treatment significantly reduced tumor growth in a B-cell lymphoma model increasing mice survival [46].

More recently a remarkable study from Gilboa's group [87] has been published in which the bivalent murine 4-1BB aptamer was conjugated with a siRNA for raptor, a key factor of the mTORC1 (mTOR complex 1). mTOR is an intracellular mediator associated with accumulation of immune-system short-living effector cells. This construct promoted mTOR1 downregulation [87]. Finally, this construct in combination with already established vaccination protocols promoted a potent memory response with cytotoxic effector functions and protective immunity in tumor-bearing mice [87]. Although the expression of new and therefore more potent antigens displays an antitumor immune response, in some cases it is not sufficient (in the majority of tumors due to its immunosuppressive microenvironment). In fact, the expression of new antigens induces a higher Treg infiltration, indicating that the combination with other antagonist aptamers towards receptors such as IL-10R, CTLA-4, PD1, or TIM-3 would be of great interest to optimize the antitumor immune responses. Moreover, we have recently published a new work that proves that the target inhibition of FOXP3 in Tregs can be achieved through CD28 aptamer-FOXP3-peptide blocking chimera [88]. As previously described by Casares et al. [89], P60 is a FOXP3 inhibitor peptide able to penetrate the membrane of Tregs and thereby to inhibit FOXP3 [89]. Due to lack of specificity, the required doses are very high; thus, we managed to conjugate this peptide with one of our CD28 described aptamers to deliver P60 to CD28-expressing cells. The CD28-targeted P60-mediated FOXP3 inhibition was able to counteract Treg immunosuppression by reducing the concentration to 0.5 *μ*M [89]. This significant reduction means that concentration can be reduced up to hundreds of times to obtain the same effect [89]. The treatment of CT26 colon carcinoma-bearing mice with the immunodominant CT26 peptide AH1 and incomplete Freund's adjuvant in the presence of 625 pmol of the CD28 aptamer-P60 chimera controlled tumor growth at a similar rate compared to that of 500 nmol of the respective P60 control [89].

## 3. Concluding Remarks

Aptamers have emerged as a great new therapeutic class of reagents very suitable for cancer immunotherapy. Several aptamers have been described to date and some of them have reached clinical grade, but we are still scratching the surface of the potential of this novel therapeutic platform. Aptamers are making for themselves room in cancer therapeutics thanks to their properties. Moreover, this new class of therapeutics has been described as a tool to deliver siRNAs or aptamers to the tumor to modulate the immune response. Aptamer platforms can be used to tackle the three major challenges in cancer immunotherapy: blockade of immunosuppressive mechanisms, activation of the agonistic immune receptors, and increase of the tumor immunogenicity.

## Figures and Tables

**Table 1:** Summary of agonistic and antagonistic aptamers used in cancer immunotherapy.

Target	Nature	Species	Function	Treatment	Reference
*Immune checkpoints*					
CTLA-4	RNA	Murine	Antagonist	Treatment for melanoma tumor	[35]
TIM-3	RNA	Murine	Antagonist	Treatment for colon carcinoma in combination with PDL-1 blockade	[36]
PD1	DNA	Murine	Antagonist	Treatment for colon carcinoma	[37]

*Cytokines*					
IL-10R	RNA	Murine	Antagonist	Treatment for colon carcinoma	[25]
		Human & murine	Not described	Not described	[26]
IL-6	DNA	Human	Antagonist	*In vitro* growth inhibition of human glioma and hepatoma	[38]
IL-6R	RNA	Human	Delivery	Not described	[39]
IL-4R	RNA	Human & murine	Antagonist	Treatment for mammary carcinoma	[40]
TNF-*α*	DNA	Human	Antagonist	*In vitro* prevention of TNF-*α*-induced apoptosis	[41]

*Immune receptors*					
4-1BB	RNA	Murine	Agonist	Treatment for mastocytoma tumor	[42]
		Human & murine	Not described	Not described	[26]
OX-40	RNA	Murine	Agonist	Dendritic cell-based vaccine adjuvant in melanoma tumor	[43]
		Human	Agonist	*In vitro* proliferation of CD4 T cells	[44]
CD28	RNA	Murine	Agonist	Idiotypic vaccine adjuvant for B-cell lymphoma tumor	[45]
			Antagonist	*In vitro* reversion of CD4 T cells proliferation	
CD40	RNA	Murine	Agonist	Targeted NMD inhibition in B-cell lymphoma tumor	[46]
			Antagonist	CD40 blockade in B-cell lymphoma tumor	
DEC205	RNA	Murine	Agonist	Adoptive transfer adjuvant in B16-OVA melanoma tumor	[47]
CD16*α*	RNA	Human	Antibody-dependent cell-mediated cytotoxicity (ADCC)	*In vitro* lysis of both human gastric and lung cancer cell lines	[48]
BAFF-R	RNA	Human	Antagonist	Targeted STAT-3 inhibition in mantle cell lymphoma tumor	[49]
